# Towards More Resilient Urban Landscapes: Optimal Sowing Season of 16 Native Mediterranean Species for Planting Designs

**DOI:** 10.3390/plants15050766

**Published:** 2026-03-02

**Authors:** Silvia Villegas-Navarro, Ana María Sánchez

**Affiliations:** 1 Real Jardín Botánico-CSIC, E-28014 Madrid, Spain; 2 Instituto de Investigación en Cambio Global, Universidad Rey Juan Carlos, E-28933 Madrid, Spain; ana.sanchez@urjc.es; 3 Dpto Biología, Universidad Rey Juan Carlos, E-28933 Madrid, Spain

**Keywords:** seed germination, native flora, Mediterranean, urban landscape, planting design, global change

## Abstract

Cities increasingly face the impacts of global change, demanding innovative approaches in species selection and management to create more adapted and resilient urban landscapes. The incorporation of native Mediterranean flora into planting design offers an opportunity for certain Mediterranean cities to achieve this by selecting species according to their ecological feasibility and aesthetic impression throughout the year. This study constitutes a first step towards understanding the germination behaviour of 16 native Iberian plant species, with potential for use in urban planting through direct seeding. Species were selected for their ecological feasibility in Mediterranean urban environments and to maximise functional diversity in growth forms, phenology, and other vegetative and reproductive traits, thereby supporting biodiversity and ecosystem resilience. Controlled germination trials were used to evaluate germination percentage and mean germination time of 16 species under temperature and light conditions that simulated autumn and spring, the main Mediterranean germination seasons, and spring-like conditions following cold stratification. The results revealed variability in seed germination among scenarios and species, indicating diverse and contrasting germination strategies. The majority of species achieved their highest final germination percentage under the autumn scenario. Germination speed was also strongly scenario-dependent, with several species completing germination within 10 days, and cold stratification reducing the mean germination time of the most responsive species. Based on these findings, a functional classification is proposed to guide the optimal sowing season. Although autumn appears to be the most favourable time for seed sowing, species-specific germination patterns must be considered to ensure successful establishment and the effective inclusion of each species in seed mixtures.

## 1. Introduction

Cities are facing unprecedented climate challenges that require long-term, innovative, and sustainable strategies able to cope with a hotter and drier urban world [1]. The new environmental conditions in cities, which are experiencing an increasing frequency and severity of global change impacts such as heat waves and droughts [2], imply an urgent need to introduce novelties in species selection and management, which will contribute to more adapted and resilient urban landscapes [3,4]. Across multiple nations, city officials and local residents are exploring options for more sustainable vegetation to replace traditional planting, such as turf grasses with meadows, flower-rich lawns [5,6,7] or prairies [8]. Furthermore, there is a growing interest in the use of native vegetation [9,10] to provide a more diverse range of environmental and social benefits [11]. Therefore, the demand for genetically and species-diverse native plant materials (seeds, plugs, sod mats, cuttings, container plants, and bare rootstocks) is expected to increase in the coming years [12]. In the case of seeds, identifying the germination requirements of each species will determine its suitability for planting designs.

The benefits provided by native vegetation in cities will be greatly reinforced through naturalistic planting design, a concept that involves combining species in ways that reflect the character they display in the wild [13]. Naturalistic and ecologically inspired designs of urban green spaces are more sustainable [14], more effective at encouraging wildlife [15], and far less expensive to manage when compared with more formal and traditional landscapes [16]. Furthermore, it also presents an opportunity to create new visual forms, changing traditional designs dominated by the homogeneous arrangement of evergreen shrubs into more diverse landscapes. Naturalistic planting designs can instead consist of a diverse array of plant growth forms, including long-flowering herbaceous plants, biennials, short-lived species, shrubs, and subshrubs. The inclusion of different life forms (trees, shrubs, herbs, etc.) also assists in providing additional niches for diverse faunal taxa [17,18] and sensitive species with more specialised requirements.

Due to their high resistance to pests, high salt tolerance, high water use efficiency and the fact that their growth patterns are well adapted to the prevailing environmental conditions of long, dry summers and extreme temperatures [19,20,21,22,23,24], Mediterranean flora species offer an effective alternative to the ornamental plants traditionally used in semiarid ecosystems. However, the design of the planting, as well as the selection and combination of plants, must differ from those employed in meadow-like plantings of cooler climates [25]. A functionally diverse combination of species, considering characteristics such as size, shape, foliage density, texture, root mass, rate of growth [26], flower shape, size and colour, number of inflorescences, phenology and resistance to water limitation in non-irrigated plantings, should be considered to achieve the desired aesthetic impression when using them in urban landscapes.

Although there is ample evidence of the benefits of incorporating Mediterranean species in planting schemes, their utilisation in urban landscapes in Spain remains limited, which mainly consists of conventional ornamental designs dominated by street trees such as *Celtis australis* L. and *Platanus acerifolia* var. *hispanica* (Mill. ex Münchh) Bean, as well as palm trees such as *Phoenix canariensis* Chabaud and *Trachycarpus fortunei* (Hook.) H. Wendl. Extensive lawns are also abundant, together with monochromatic mass planting of evergreen shrubs such as *Ligustrum japonicum* Thunb., *Prunus laurocerasus* L., *Viburnum tinus* L. and *Buxus sempervirens* L. and seasonal bedding plants. The scarce attention given to alternative naturalistic planting designs using native flora with diverse life forms is especially striking, given the high species richness of the flora of the Iberian Peninsula and Balearic Islands, with more than 189 families, 1266 genera and 6176 species [27]. Although they all constitute a huge pool of species adapted to the environmental conditions characteristic of the Mediterranean region [28], there is little practical information regarding their requirements and the conditions needed to include them in Mediterranean urban landscapes.

As natural planting designs have increased and evolved, there has been a growing interest in establishing vegetation using seed mixtures for direct seeding. This is because species established by seeding require less resource input for establishment and subsequent management than more conventional plantings using nursery-grown plants. Because it avoids the energy, infrastructure, substrate, irrigation, and labour associated with nursery production, it also allows much larger areas to be created [13,16]. However, seed germination is a complex physiological process subjected to environmental cues, mainly temperature and soil moisture [28,29,30]. Species that reproduce by seed tend to be associated with habitat conditions, such as light availability and temperature regime, with low temperatures limiting germination and day length influencing seedling establishment [31,32]. In the Mediterranean, water limitation also shapes seed germination phenology to maximise recruitment during the season of highest water availability [33]. Furthermore, many species exhibit seed dormancy, which is a plant strategy where the seeds do not germinate, although environmental conditions are favourable for germination [29]. Therefore, knowing the germination behaviour of seeds must be the first step to the successful use of Mediterranean species in natural planting, especially for establishing them by direct seeding with seed mixtures. The design of these mixtures should consider each species germination percentage and germination timing. These two factors will greatly determine the resulting community, as they are closely related to the species ability to establish and compete in the seed mixture after sowing [28]. Furthermore, germination can vary among seasons, and thus, germination trials should be performed to determine the optimal seeding density and sowing periods [34]. At present, there is a lack of available information regarding the germination and optimal sowing periods of most of the native species from the Iberian Peninsula. This absence of data represents a significant impediment to the promotion and use of them by authorities and residents in cities.

The present study constitutes a first step towards understanding the germination behaviour of 16 native Mediterranean species, belonging to six families, from the Iberian Peninsula, information that is relevant to enhance their use in urban planting designs in Mediterranean climates by direct seeding. Specifically, the study has the objective to characterise the germination response under contrasted environmental conditions of the 16 species selected for their ecological and aesthetic attributes. Germination behaviour under autumn and spring conditions has been compared, when seed germination is more likely to occur naturally in the Mediterranean Basin. To achieve this, the effects of various temperature and light conditions and cold stratification on seed germination under controlled laboratory conditions have been tested. The results clarify the suitability of the study species for use in seed mixtures and provide practical information for optimising their use in natural designs. Moreover, the germination trials conducted will serve as a standardised, simple protocol that can be applied to the characterisation of the germination of many other Mediterranean species.

## 2. Results

All the studied species presented a final germination percentage (FGP) of more than 10% in at least one of the tested scenarios, except *Linaria clementei*, which exhibited a maximum FGP of around 10%. For this reason, the effects of the three experimental scenarios on the FGP and mean germination time (MGT) in days were not assessed for *Linaria clementei* (Table 1).

For most species, significant differences in germination responses across scenarios were observed, with the exception of *Centaurea barrasii*, *Thymus membranaceus* and *Helianthemum squamatum*, which showed no significant variation (*p* > 0.05) according to the Likelihood Ratio Chi-Square test.

Cold stratification induced seed germination during the stratification period in *Cynara humilis* (84%), *Margotia gummifera* (47%) and *Magydaris panacifolia* (81%), while the remaining seeds exhibited fungal contamination. As germination occurred during cold stratification rather than after transfer to spring-like conditions, the calculation of FGP and MGT was not possible for these three species under the cold stratification followed by the spring-like condition scenario (C-S). The FGP and the MGT could not be determined either under spring-like conditions (C-S) for *Helianthemum squamatum* and *Bupleurum rigidum* because none of their seeds germinated after cold stratification due to severe fungal contamination. In the case of *Centaurea clementei* and *Centaurea prolongoi*, some of the seeds germinated during cold stratification. However, the remaining seeds were tested under the spring-like conditions after cold stratification (C-S). Similarly, *Bupleurum rigidum* and *Margotia gummifera* were excluded from these comparisons, as FGP under the spring scenario (S) was <10% and no germination occurred under spring-like conditions (C-S) (Table 1).

For species in which post-stratification germination at 20 °C could be quantified (C-S), FGP was significantly reduced compared to spring (S), including *Centaurea clementei*, *Cynara baetica*, *Phlomis lychnitis* and *Thymus longiflorus*. In contrast, *Moricandia arvensis* benefited from cold stratification, exhibiting the highest FGP under spring-like conditions (C-S). *Smyrnium olusatrum* also performed better at 20 °C after cold stratification than under spring conditions (S) (Table 1).

The majority of species achieved the highest FGP under the autumn scenario (A). Near-complete germination (100%) was observed for *Centaurea barrasii*, *Centaurea clementei*, *Centaurea prolongoi*, *Magydaris panacifolia*, *Phlomis crinita*, *Phlomis lychnitis*, *Thymus longiflorus* and *Thymus membranaceus* (Table 1). An FGP of more than 75% was observed in *Cynara baetica*, *Cynara humilis* and *Margotia gummifera*.

Under the S condition, seven species also reached 100% germination: *Centaurea barrasii*, *Centaurea clementei*, *Magydaris panacifolia*, *Phlomis crinita*, *Phlomis lychnitis*, *Thymus longiflorus* and *Thymus membranaceus*. An FGP of approximately 70% was observed in *Centaurea prolongoi* and *Cynara baetica*. In contrast, *Margotia gummifera*, *Bupleurum rigidum* and *Smyrnium olusatrum* exhibited significantly lower FGP under S (3–19%) compared to A. Pairwise comparisons between S and A revealed no significant differences in the FGP for *Centaurea barrasii*, *Centaurea clementei*, *Centaurea prolongoi*, *Cynara baetica*, *Helianthemum squamatum*, *Magydaris panacifolia*, *Phlomis crinita*, *Phlomis lychnitis*, *Thymus longiflorus* and *Thymus membranaceus* (Table 1).

**Figure 1:**
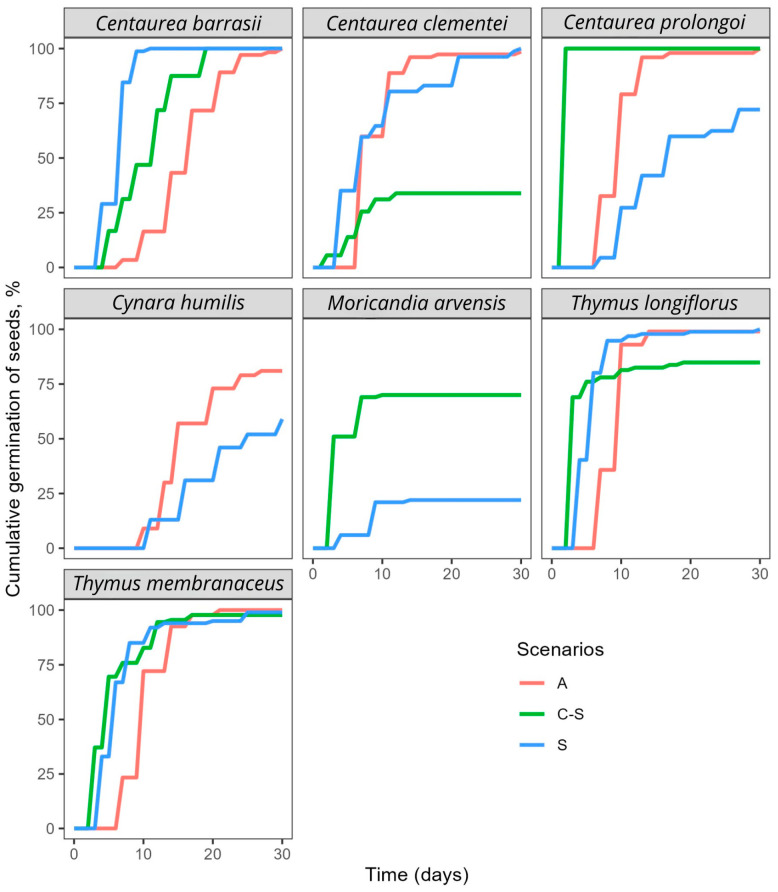
Cumulative germination curve of the study species under autumn (A), spring-like conditions after cold stratification (C-S), and spring (S) over 30 days.

**Figure 2:**
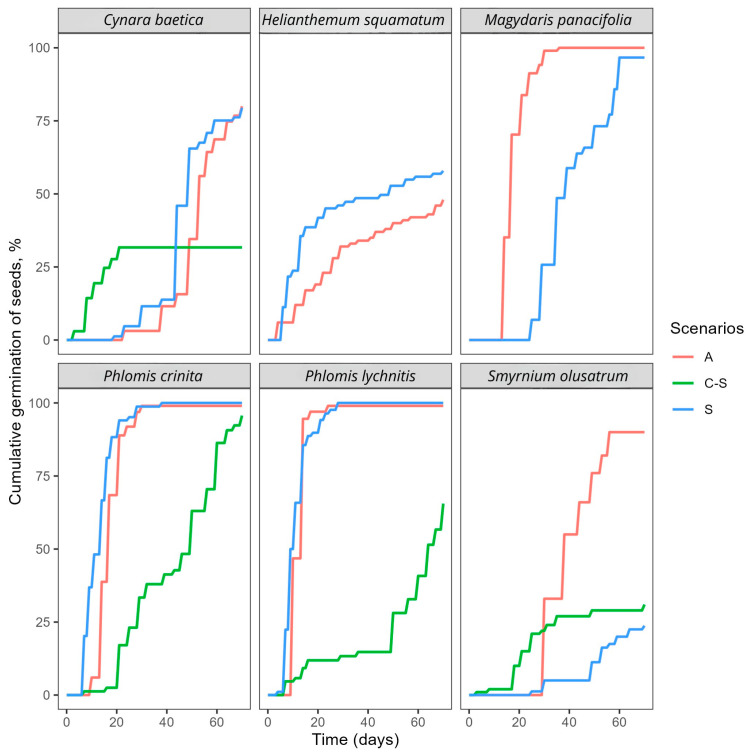
Cumulative germination curve of the study species under autumn (A), spring-like conditions after cold stratification (C-S), and spring (S) over 70 days.

Regarding MGT, cumulative germination curves indicated that germination speed was significantly affected by the scenarios in most species (Figure 1 and Figure 2).

For species such as *Moricandia arvensis* and *Thymus longiflorus*, germination was completed in approximately 10 days or fewer across all scenarios. The MGT was observed to increase under A compared to others in *Centaurea barrasii*, *Helianthemum squamatum*, *Thymus longiflorus* and *Thymus membranaceus*. Conversely, cold stratification led to a reduction in the MGT in several species, with the exception of *Phlomis crinita* and *Phlomis lychnitis*, which exhibited a significant increase (Table 1; Figure 2).

Under the spring condition (S), MGT was greater in *Centaurea clementei*, *Centaurea prolongoi*, *Cynara humilis*, *Magydaris panacifolia*, *Moricandia arvensis* and *Smyrnium olusatrum*. Pairwise comparisons between spring (S) and autumn (A) revealed no significant differences in MGT for *Centaurea clementei*, *Cynara baetica*, *Phlomis crinita*, *Phlomis lychnitis* and *Smyrnium olusatrum* (Table 1; Figure 2).

Considering all species, the autumn (A) scenario was the most favourable treatment, benefiting 75% of the species studied, whereas the spring (S) scenario and spring-like conditions after cold stratification (C-S) were optimal for 18.75% and 6.25% of species, respectively.

## 3. Discussion

This study presents empirical evidence on the seed germination strategies of 16 native Mediterranean plant species from the Iberian Peninsula, with promising implications for their use in naturalistic planting designs through direct seeding (Table 1).

The provided results are of particular importance, as the success of such applications relies heavily on the germination response of each species, both in terms of quantity (FGP) and time (MGT). Understanding species-specific germination requirements is essential for optimising sowing strategies, including the appropriate timing of seeding and the number of seeds required to ensure the successful establishment of all species within the seed mixtures. In addition to germination success, germination speed must also be considered. The time required for each species to germinate will determine a hierarchy in terms of access to space and resources, which in turn will affect plant establishment, growth, survival and the dynamic of the resulting community [35].

The results demonstrate successful germination of more than 10% in all species in at least one of the tested scenarios, except *Linaria clementei*. However, variability was observed in both FGP and MGT across the scenarios (Table 1), which reflects the contrasting strategies performed by the different species to germinate.

Altogether, this results in a diverse array of germination strategies [33] or regeneration niches [36,37] that have to be considered in order to design a successful planting procedure.

Based on data from standardised germination experiments, we propose a functional classification of species according to their optimal sowing season and germination outcomes in terms of FGP and MGT: (1) species best sown in autumn, (2) those more suited to spring sowing, and (3) species requiring cold stratification for optimal germination outcomes. The classification offers a practical time framework for incorporating them into seed mixtures for planting designs (Table 2).

### 3.1. Autumn: Optimal Season for Germination

The study presents evidence indicating autumn-like conditions as the most successful season for seed germination in the majority of studied species (Table 1; Figure 1 and Figure 2). This aligns with recent research that identified autumn as the most common germination period for Mediterranean species [33] and reveals the importance of Mediterranean macroclimatic conditions in shaping the species germination niches.

More closely related species, particularly those within the Apiaceae (*Bupleurum rigidum*, *Magydaris panacifolia*, *Margotia gummifera* and *Smyrnium olusatrum*) and Asteraceae families (*Centaurea clementei*, *Centaurea prolongoi*, *Cynara baetica* and *Cynara humilis*) showed similar germination responses and superior outcomes in values of FGP, MGT or both under the A condition (Table 1; Figure 1 and Figure 2). Notably, stratification at 4 °C also stimulated germination during this period for species belonging to both families, suggesting that, rather than coming out of dormancy, cold-moist conditions provide suitable environmental cues such as low temperature, darkness, and soil moisture for seed germination, factors inherently prevalent during autumn and winter in Mediterranean climates. The species included in the Lamiaceae family (*Phlomis crinita*, *Phlomis lychnitis*, *Thymus membranaceus* and *Thymus longiflorus*) showed similar results in FGP under the A and S conditions, which leads us to believe in some flexibility in sowing time for these taxa. The results are also consistent with previous studies of *Thymus* species from semiarid regions, where germination of fresh seeds exhibited high and rapid germination within a wide range of temperatures, particularly those similar to the seasons where rainfall is concentrated. However, high temperatures can induce an innate conditional physiological dormancy when water stress severely threatens the survival of seedlings [38]. A similar phenomenon was observed in other species of *Phlomis*, where FGP decreased at high constant temperatures of 25 °C and low temperatures of 5 °C [39]. This was also observed in our study in both *Phlomis*, where, after cold stratification, the MGT was higher and the FGP lower (Table 1).

On the other hand, MGT was influenced by the three scenarios in both *Thymus* species (Figure 1). Although differences were observed, they were not large enough to favour spring sowing over autumn. Because autumn germination is the most frequent germination timing under Mediterranean conditions [28,33,40,41], seedlings reach summer with a more developed root system than those with spring germination [42]. However, there are inherent risks associated with sowing seeds of the studied species in autumn. If they are unable to germinate within an adequate timeframe, they can suffer an important predation risk while in the soil [43] and of fungal contamination, especially species belonging to the Apiaceae family. Additionally, species such as *Smyrnium olusatrum*, *Phlomis crinita* and *Phlomis lychnitis* may enter into a form of dormancy due to prolonged exposure to temperatures below 4 °C during winter, resulting in a decrease in the FGP the following spring and an increase in the MGT (Figure 2), as observed in the experimental results. These species exhibit a significant decline in germination speed following exposure to cold stratification. Furthermore, young seedlings can suffer from below-zero temperatures common in continental winters. However, although some mortality cannot be disregarded, previous experimental studies with Mediterranean annual plants have shown a high resistance to cold temperatures [44].

### 3.2. Spring: Opportunities for Rapid Germination

In spring, increasing temperatures benefited some species, particularly in terms of germination speed. *Centaurea barrasii* exhibited a similar FGP under the A and S conditions, but with a notable increase in germination speed under the S condition. This is consistent with observations in *Centaurea* species, indicating their tolerance to a broad temperature range, with optimal germination between 12.5 and 25 °C [45]. Consequently, spring sowing is recommended due to the superior germination speed observed. Similarly, for *Helianthemum squamatum*, the differences are evident in both the FGP and the MGT, with superior results observed under the S condition, which is in accordance with observed germination timing in the field [46], and with the ability of seedlings to successfully thrive under summer conditions, at least when growing in the gypsum soils to which this species is strictly restricted [41]. Furthermore, gypsum scrublands are among the least productive Mediterranean habitats due to the physical and chemical restrictions these soils impose on plant development [47]. This means that the low perennial cover has less interference with the standing plants and allows the establishment of new seedlings during the spring [48].

Under planting conditions, it will be important to ensure that germination occurs rapidly when sowing both species during the spring. This will allow the seedlings to establish adequately before the onset of the summer drought period. Rapid germination of each species will facilitate superior establishment, but the number of seeds of each species must be calibrated to avoid competition from early-establishing species [49]. This is because, in the field, the remaining seeds with a slower germination speed will gradually lose viability as fast germinating species establish and begin to grow [35]. This leads to increased competition for space, light and resources, which in turn reduces the FGP, emergence and establishment success.

### 3.3. Cold Stratification Followed by Spring Sowing

Certain species, notably *Moricandia arvensis*, demonstrated superior outcomes in the FGP and the MGT following stratification at 4 °C, attributed to the disruption of a physiological dormancy. The results are also not unexpected, as *Moricandia arvensis* exhibits ruderal characters, rapid growth and floral plasticity that allows the same individual plants to reproduce successfully in a wide range of environments in semiarid and arid ecosystems. The same plants can start to flower in early spring and continue throughout the summer and even during autumn, producing a similar amount of seeds from two types of flowers under field conditions [50,51]. This strategy enables the plants to produce high levels of viable seeds during periods when other species are inactive, while delaying some germination until late spring. This behaviour is also associated with the dormancy of a high level of seeds as a conservative strategy in high-risk environments such as disturbed ground.

Species replacement considerations

*Linaria clementei* shows the least successful FGP species under all three tested scenarios. Although the seeds were visually in good condition and water-permeable, the lack of germination may be attributed to exposure to temperatures and light above or below those required for germination, or to some form of seed dormancy or poor seed quality. In any case, it is very difficult to assess in this experiment due to the small size and light weight of the seeds. It is recommended that alternative methods, such as detailed inspection of embryo development and dormancy, be employed to further investigate it.

Taking into account the results obtained, it is proposed that *Linaria clementei* should be replaced by another perennial species within the family Scrophulariaceae, given that its flowers present a nectar spur that attracts bees, bee flies, moths, and beetles [52]. This would serve to introduce plant functional diversity, increasing diversity in pollinators. *Linaria triornithopora* (L.) Cav., which is widely distributed in the northwest of the Iberian Peninsula from 0 to 2000 metres, is proposed as a replacement. It grows in thickets and forest clearings, on slopes and in damp banks on limestone or siliceous substrates, and flowers from March to September. *Linaria aeruginea* (Gouan) Cav. also has a wide distribution on the peninsula, where it grows on limestone rocks, scree, and in scrubland. It can be found growing at altitudes ranging from 150 to 3300 metres, with its flowers blooming from April to July. *Linaria alpina* (L.) Mill. is present in the northern and central mountain ranges of the Iberian Peninsula, growing at altitudes between 250 and 3300 metres, and it flowers from April to September [53]. In the same family, *Antirrhinum graniticum* Rothm. can also be used, which is widely distributed across the Iberian Peninsula from 300 to 1300 metres. It grows in a variety of habitats, including rock fissures and landings, stony disturbed ground, roadsides, rubble, walls of gypsum, slate, schist, and granite. Its blooming period extends from April to July [54].

In any case, further studies should also explore the success of these 16 species in terms of germination, emergence, survival and growth in field conditions. Future studies should also explore other Mediterranean species under varying temperature and photoperiod conditions to expand the plant palette for planting design. This work will be instrumental in achieving resilient, low-input, and ecologically functional urban green spaces in the Mediterranean climate.

## 4. Materials and Methods

**Figure 3:**
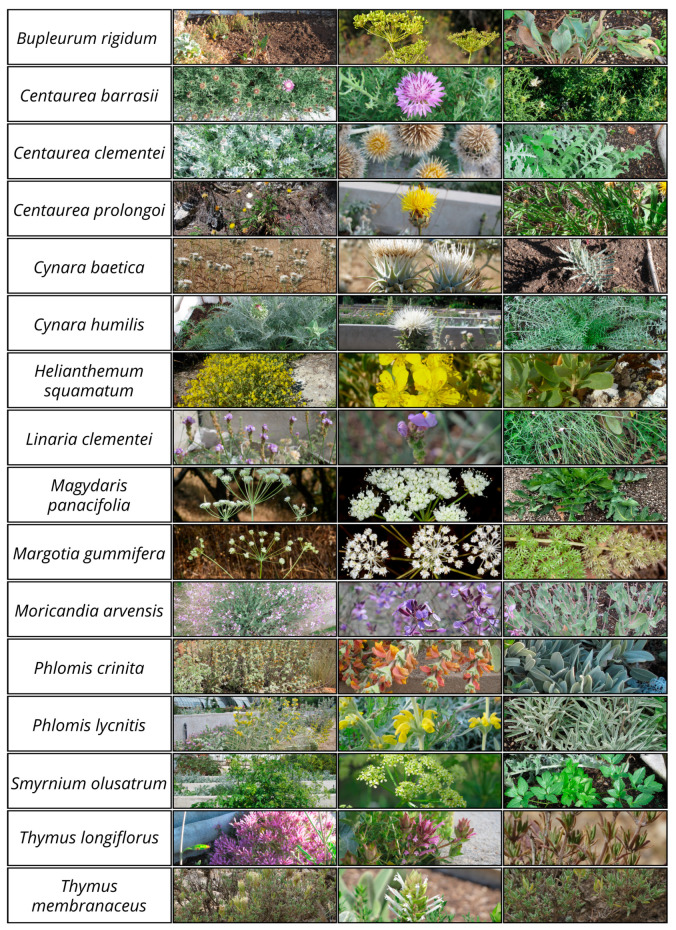
Visual attributes of the species. The first column corresponds to the life form, the second column corresponds to the flowers, and the third column corresponds to the leaves.

A total of 16 native species from the Iberian Peninsula were selected for use in planting design in central Spain. The nomenclature used for the taxa is in accordance with that set out in *Flora iberica* [27] and Plants of the World Online [55] (Figure 3).

Species selection was based on their ecological feasibility for Mediterranean cities, defined as their ability to survive, establish, and perform under the Mediterranean climatic conditions, including those experiencing cold winters. All the selected species geographical distribution ranges occur in central and southern Spain, at altitudes ranging from 90 to 1200 metres, and the seeds have been collected from locations where climatic conditions are at least as harsh as those typically occurring in central Spain, where the experimental sowing will be based upon this germination characterisation.

Additionally, regarding the climatic criteria, the ability of the study species to thrive under suboptimal soil conditions in terms of nutrients, physical structure, and depth has been considered. All of them are highly stress-tolerant species and able to develop even under poor soil conditions, which will guarantee an attractive and aesthetically pleasing performance in a wide range of urban situations.

Among the huge number of species that could have fulfilled the above criteria, we selected a group of species that maximises the functional diversity in terms of growth forms, phenology, and other vegetative and reproductive (flowers and fruits) traits (Figure 3).

Enhancing functional diversity [17,18] will enable the planting design to better support a functionally complex and diverse animal community, which will provide the design with a higher resilience against pests and diseases and allow it to provide more and better ecosystem services. Alongside the previous ecological criteria, the desired aesthetic impression throughout the year was taken into account by considering various characteristics, including size, shape, density of foliage, texture, colour and abundance of flowers, phenology, and life form, as referred to in *Flora iberica* [27]. Furthermore, consideration was given to the summer texture and structure of the leaves and inflorescence (Table 3; Figure 3).

After taking all these criteria into consideration, the resulting set of species constitutes a diverse subset representation of the Mediterranean flora suitable for urban planting design, both in terms of taxonomy (comprising six different families) and ecology; they occur in different types of shrubby and forest communities (Figure 3; Supplementary Materials).

Seed collection

Mature seeds were collected between May and September 2019 and 2020 from native sources at the time of natural dispersal. Prior to the commencement of seed collection, the necessary permits were obtained from regional authorities. For each species, plants in the population were randomly selected for seed collection, with the objective of sampling the variation and genetic diversity present without endangering the population.

Following collection, the seeds were distributed on trays and dried in a laboratory setting at a temperature of 20–22 °C and a relative humidity of 15–20%. Subsequently, the number of seeds per one gram was determined by three repeated counts, and the seeds were then stored for two weeks in paper bags at room temperature. All germination tests and stratification treatments were initiated simultaneously for each species; this ensured that storage duration and conditions were consistent across species.

Germination test

To achieve the objectives, one experiment with three treatments was conducted. Seeds were incubated (1) at a constant temperature of 10 °C under a 12 h light/dark photoperiod to simulate the average temperature and photoperiod in autumn (A), (2) at a constant temperature of 20 °C under a 16 h light/8 h dark photoperiod, mimicking the average temperature and photoperiod in spring (S), and (3) after prior stratification for 21 days at 4 °C in moist sand in the absence of light, and subsequent incubation at a constant temperature of 20 °C under a 16 h light/8 h dark photoperiod (C-S). Light and temperature reference values were obtained from a Spanish Meteorological Agency (AEMET) station in Madrid. For each scenario, 10 independent replicates were used per species, with each replicate consisting of a 90 mm plastic Petri dish containing 10 seeds on a double layer of filter paper moistened with distilled water, totalling 100 seeds per species/treatment. In the case of *Centaurea prolongoi*, 5 replicates of 10 seeds were used for each scenario, due to the unavailability of seeds. The paper was saturated at the beginning of the experiment and subsequently maintained at a saturated state through regular watering. The experiments were conducted in two Friocell 222 incubators (MMM Medcenter Einrichtungen GMbH, Planegg, Munich, Germany), equipped with cool-white fluorescent tubes (Philips TL-D 18 W/840) located at the door. Cold stratification was achieved by maintaining the seeds in a moist sand environment in the absence of light, within Petri dishes wrapped with a double layer of aluminium foil at 4 °C for 21 days [29]. The average duration of the stratification period was established based on the observation that in previous trials, some seeds germinated prematurely or exhibited signs of fungal contamination when subjected to longer stratification periods. Given the different germination conditions of various species and the evidence from previous laboratory trials indicating that some species require more time to germinate than others, the study was divided into two incubation periods assigned previously by species: (1) seeds of faster germinating species were incubated for 30 days, following Baskin and Baskin [29], and (2) seeds of slower germinating species were incubated for 70 days, to allow sufficient time to germinate. These two incubation periods correspond to different species groups and do not represent a sequential prolongation of the same experiment (Table 4).

The emergence of a visible radicle from the covering structures of the seeds [29,30] was used as the criterion for determining the germination status. Germinated seeds were counted and removed every 2–3 days, and the filter paper was moistened with distilled water if necessary, taking care to avoid contact between the water and the seeds. At the conclusion of the test, seed viability was evaluated using the cut test [29]. Seeds were sectioned to inspect the embryo: those exhibiting indications of fungal growth, a soft/discoloured texture, or a collapse when squeezed were considered non-viable. Conversely, seeds with firm, white, and turgid embryos were confirmed as viable. All germination parameters were calculated from viable seeds.

Statistical analysis

FGP and MGT were calculated separately under autumn (A), spring-like conditions after cold stratification (C-S), and spring (S). FGP represents the proportion of seeds that have germinated out of the total viable seeds used in each replicate at the conclusion of the germination test. MGT represents the mean number of days the seeds spent to germinate [56]. FGP and MGT were calculated using GerminaR, including the median and standard deviation [57].

The effect of the three different scenarios on the germination response of each species was estimated using generalised linear models (GLMs). The conditions (autumn (A), spring (S), and spring-like conditions after cold stratification (C-S)) were designated as categorical fixed factors (the explanatory variable), with FGP and MGT as the response variables. Model significance was assessed using likelihood ratio tests based on the Chi-Square distribution, and statistical inference was based on the associated probability values Pr(>Chisq).

To assess the FGP, a binomial family with a logit link function was used, with germination considered as a binary variable, given that seeds may or may not germinate. A One-Way ANOVA type I test was conducted on the model. The three different scenarios’ effects on the MGT were estimated using a GLM, in terms of the quasi-Poisson family. A One-Way ANOVA type I test was applied to the model in accordance with the methodology proposed by Cayuela and de la Cruz [58].

The models were only performed for scenarios with an FGP > 10%. The D-squared (D^2^) value was calculated for each GLM in order to assess the model’s fit to the data, using the formula (null deviance-residual deviance)/null deviance, as proposed by Sánchez et al. [59].

The statistical analysis was conducted using R software, statistical version 4.3.2 [60].

## 5. Conclusions

The study provides valuable evidence to support the direct seeding of Mediterranean species in naturalistic planting designs in urban landscapes, particularly in habitats where soil disturbance is limited and environmental conditions are semi-natural. Direct seeding is especially suitable for extensive planting areas, such as road roundabouts, park margins, planting beds, urban meadows and low-management green infrastructure, where irrigation and maintenance inputs are minimal.

Although autumn clearly emerges as the best time for seed sowing, specific studies such as this one allow us to identify unique germination patterns that must be considered for the successful inclusion of certain species in seed mixtures in designs. Aligning sowing periods with species-specific germination conditions enables us to maximise establishment success and long-term resilience.

Incorporating seasonally appropriate and phylogenetically diverse species into planting designs will enhance biodiversity, reduce maintenance requirements, and promote ecosystem functionality in Mediterranean urban environments.

## Figures and Tables

**Table 1:** Final germination percentage (FGP) and mean germination time (MGT) in days; mean values and one standard deviation (SD) for 16 species under the three scenarios of autumn A (10 °C, 12 h light/dark), spring S (20 °C, 16 h light/8 h dark) and spring-like conditions after cold stratification C-S (4 °C for 21 days in dark moist sand, followed by 20 °C, 16 h light/8 h dark). The results of generalised linear models (GLMs) estimating the impact of the three scenarios on FGP and MGT for each species are outlined. A binomial family with a logit link function and a One-Way ANOVA type I test was applied to the models for FGP. A quasi-Poisson family One-Way ANOVA type I test was applied for MGTl. df = degrees of freedom; D = deviance. *p*-values are shown in the Pr(>Chisq), statistical significance was set at *p* < 0.05, and ---- means no values available. The rationale for using these two statistical approaches is detailed in the Section 4.

Species	FGP (%)									MGT (Days)								
	Autumn		Cold Spring		Spring		GLM			Autumn		Cold Spring		Spring		GLM		
	Mean	SD	Mean	SD	Mean	SD	df	Pr(>Chisq)	D	Mean	SD	Mean	SD	Mean	SD	df	Pr(>Chisq)	D
*Bupleurum rigidum*	62.98	23.47	-----	----	2.69	5.66	2	-----	-----	57.41	7.41	-----	-----	24.00	12.73	2	-----	-----
*Centaurea barrasii*	100	0	100	0	100	0	2	1	-----	16.55	2.07	10.82	4.50	6.46	0.94	2	<0.001	0.73
*Centaurea clementei*	98.57	4.52	33.90	25.43	100	0	2	<0.001	0.84	9.11	1.30	5.92	2.22	9.6	5.09	2	<0.05	0.24
*Centaurea prolongoi*	100	0	100	0	72.12	16.17	1	<0.001	0.78	10.07	1.93	2.00	0	14.79	1.02	1	<0.001	0.94
*Cynara baetica*	80.00	15.02	31.69	17.90	79.48	17.18	-----	<0.001	0.60	51.42	2.86	13.97	4.92	45.3	5.13	-----	<0.001	0.92
*Cynara humilis*	81.00	12.87	-----	-----	59.00	16.63	-----	<0.001	0.33	15.90	2.57	-----	-----	19.48	3.30	-----	<0.001	0.29
*Helianthemum squamatum*	48.00	6.32	-----	-----	57.89	22.83	1	0.22	0.06	30.01	11.42	-----	-----	18.45	8.09	1	<0.001	0.29
*Linaria clementei*	11.00	11.00	4.00	6.99	9	12.87	2	-----	-----	22.00	4.90	19.83	6.45	19.05	5.76	2	-----	-----
*Magydaris panacifolia*	100	0	-----	-----	96.67	10.53	2	<0.05	0.32	18.15	2.05	-----	-----	40.73	9.49	2	<0.001	0.81
*Margotia gummifera*	80.62	20.69	-----	-----	6.96	18.05	-----	-----	-----	21.03	2.76	-----	-----	30.00	0	-----	-----	-----
*Moricandia arvensis*	5.00	7.07	70.00	21.60	22.00	12.29	2	<0.001	0.61	12.75	2.50	4.14	0.93	7.71	2.04	2	<0.001	0.59
*Phlomis crinita*	99.00	3.16	95.65	7.07	100	0	1	<0.05	0.40	17.48	2.87	43.04	13.57	13.32	2.09	1	<0.001	0.83
*Phlomis lychnitis*	99.00	3.16	65.52	28.28	100	0	1	<0.001	0.69	12.37	1.34	49.70	8.87	11.62	1.98	1	<0.001	0.95
*Smyrnium olusatrum*	90.00	10.54	31.00	20.25	19.00	21.32	2	<0.001	0.69	39.83	5.50	24.49	10.05	45.93	14.84	2	0.11	0.11
*Thymus longiflorus*	99.00	3.16	84.87	14.44	100	0	2	<0.001	0.57	9.15	1.05	4.05	1.63	6.07	1.33	2	<0.001	0.71
*Thymus membranaceus*	100	0	97.75	4.78	99.00	3.16	2	0.24	0.24	10.73	1.90	5.91	2.04	7.08	2.02	2	<0.001	0.53

**Table 2:** Summary of the studied species, classified according to their optimal sowing seasons in areas under Mediterranean climatic conditions, including the number of seeds per gram for each species (N° seeds/g) and the final germination percentage (FGP, %).

Optimal Sowing Seasons	Family	Species	N° Seeds/g	FGP (%)
Autumn	Asteraceae	*Centaurea clementei*	96	100
	Asteraceae	*Centaurea prolongoi*	102	100
	Asteraceae	*Cynara humilis*	13	81
	Asteraceae	*Cynara baetica*	41	80
	Lamiaceae	*Phlomis crinita*	101	99
	Lamiaceae	*Phlomis lychnitis*	110	99
	Lamiaceae	*Thymus longiflorus*	5925	100
	Lamiaceae	*Thymus membranaceus*	4348	99
	Apiaceae	*Bupleurum rigidum*	483	63
	Apiaceae	*Magydaris panacifolia*	316	100
	Apiaceae	*Margotia gummifera*	61	80
	Apiaceae	*Smyrnium olusatrum*	26	90
Spring	Asteraceae	*Centaurea barrasii*	157	100
	Cistaceae	*Helianthemum squamatum*	2735	58
Spring, previous cold stratification	Brassicaceae	*Moricandia arvensis*	3223	70

**Table 3:** Life forms and selection attributes of the study species.

Species	Life Form	Height (cm)	Leaf Colour/Texture	Flower Structure/Corolla Colour	Flowering Months	Special Traits	Functional Objectives
*Bupleurum rigidum* L.	Perennial herb	Up to 150	Green-blue/stiff, glaucous, coriaceous	1–10 flowers per terminal and lateral umbels. Actinomorphic. Yellow-green	VI–XI	Multibranched, extended flowering period. It provides nectar and pollen for pollinating insects	Biodiversity
*Centaurea prolongoi* Boiss. ex DC.	Biennial or short-lived	Up to 10–30	Green/stiff, rough	Inflorescence in radiant capitulum. Inner florets hermaphrodite, outer sterile. Actinomorphic. Pink	IV–VI	It creates a dense ground cover	Reducing evaporation
*Centaurea clementei* Boiss. ex DC.	Perennial herb	Up to 30–50	Silver-grey/velvety, tomentose	Inflorescence in radiant capitulum. Inner florets hermaphrodite, outer sterile, as long as the inner ones. Involucre 25 mm in diameter, globose. The outer florets as long as the inner ones. Actinomorphic. Yellow	IV–VII	Enlarged outer florets, serrated tomentose leaves	Aesthetic
*Centaurea barrasii* Pau	Perennial herb	Up to 20–50	Purple-green/subglabrous, smooth	Inflorescence in radiant capitulum. Inner florets hermaphrodite, outer sterile. Involucre 15–18 mm in diameter, ovoid. Actinomorphic. Florets yellow-orange	III–VIII	Enlarged outer florets	Aesthetic
*Cynara baetica* (Spreng.) Pau	Perennial herb	Up to 70	Glabrous above, white tomentose beneath. Prickly/silver-grey	Inflorescence in radiant capitulum, solitary and very large. Ovoid involucre, surrounded by strong spiny bracts. White	VI–IX	Globose white flower heads and increased plant height	Biodiversity
*Cynara humilis* L.	Perennial herb	Up to 15–80	Glabrous above, white tomentose beneath. Prickly/grey-green	Inflorescence in radiant capitulum, solitary and very large. Ovoid involucre, surrounded by strong spiny bracts. Actinomorphic. Purplish-blue	III–VIII	Globose purple flower heads and increased plant height	Drought resistance
*Helianthemum squamatum* (L.) Dum. Cours.	Subshrub	Up to 10–30	Lanceolate, smooth, succulent/green	Inflorescence branched from base into usually 3 long-pedunculate, dense, more or less capitate cymes. Actinomorphic. Yellow	IV–VII	It forms a yellow ball-shaped bloom during flowering	Aesthetic
*Linaria clementei* Haens.	Biannual or short-lived	Up to 80–150	Linear succulent/green	Raceme subcapitate. Zygomorphic. Purple	IV–XI	Upright growth habit with linear leaves and a large number of flowers. Extended flowering period	Biodiversity
*Magydaris panacifolia* (Vahl) Lange	Perennial herb	Up to 100–250	Smooth/dark green	Umbels with 10–30 rays, pubescens. Actinomorphic flowers. White	V–VI	It provides nectar and pollen for pollinating insects	Biodiversity
*Margotia gummifera* (Desf.) Lange	Perennial herb	Up to 120	Smooth/purple-green	Umbels with 8–20 rays, pubescens. Actinomorphic flowers. White	VII–IX	It provides nectar and pollen for pollinating insects	Biodiversity/aesthetic
*Moricandia arvensis* (L.) DC.	Biannual or perennial herb	Up to 65	Smooth/grey-green	Raceme with 10–20 large, showy flowers, becoming lax. Actinomorphic flowers. Purple-white	II–XI	It has a prolonged blooming period and exhibits rapid growth. Lower leaves obovate, upper cauline leaves acute	Biodiversity/drought resistance
*Phlomis crinita* Cav.	Perennial herb	Up to 70	Velvety, coriaceous/grey-green	Vertillaster inflorescence with 6–10 flowers. Zygomorphic. Orange-brownish-yellow	IV–VIII	It creates a ground cover with lanceolate leaves, stelate–lanate. Attract pollinators	Biodiversity/reducing evaporation
*Phlomis lychnitis* L.	Perennial herb	Up to 65	Velvety/grey-green	Vertillaster inflorescence with 6–10 flowers. Zygomorphic. Yellow	III–VIII	It creates a ground cover with linear leaves. Attract pollinators	Biodiversity/reducing evaporation
*Smyrnium olusatrum* L.	Perennial herb	Up to 150	Smooth/dark green and shiny	Compound umbel inflorescence with 3–18 rays. Flowers hermaphrodite, actinomorphic. Yellow-green	II–VI	It provides nectar and pollen for pollinating insects	Biodiversity
*Thymus longiflorus* Boiss.	Subshrub; short chamaephyte	Up to 10–30	Succulent/green	Inflorescence up to 2.5 cm, subglobose. Zygomorphic. Pink-purple	V–VII	Aromatic linear leaves and prominent bracts on the inflorescences. Extended flowering period	Biodiversity/drought resistance/aesthetic
*Thymus membranaceus* Boiss.	Subshrub; short chamaephyte	Up to 10–30	Succulent/green	Inflorescence up to 2.5 cm, subglobose. Zygomorphic. White	IV–VIII	Aromatic linear leaves and prominent bracts on the inflorescences	Biodiversity/drought resistance/aesthetic

**Table 4:** Summary of the seed germination test conditions, with temperatures and photoperiods. The presence of a (+) or a (−) indicates whether the test was developed or not, respectively. Seeds subjected to the cold-spring treatment had a stratification period of 21 days at 4 °C.

Treatment	Spring (S)	Cold Spring (C-S)	Autumn (A)	
Species	20 °C 16 h Light/8 h Dark	20 °C 16 h Light/8 h Dark	10 °C 12 h Light/12 h Dark	Duration of the Germination Test (Days)
*Centaurea barrasii*	+	+	+	30
*Centaurea clementei*	+	+	+	30
*Centaurea prolongoi*	+	+	+	30
*Cynara humilis*	+	−	+	30
*Linaria clementei*	+	+	+	30
*Margotia gummifera*	+	−	+	30
*Moricandia arvensis*	+	+	+	30
*Thymus longiflorus*	+	+	+	30
*Thymus membranaceus*	+	+	+	30
*Bupleurum rigidum*	+	−	+	70
*Cynara baetica*	+	+	+	70
*Helianthemun squamatum*	+	−	+	70
*Magydaris panacifolia*	+	−	+	70
*Phlomis crinita*	+	+	+	70
*Phlomis lychnitis*	+	+	+	70
*Smyrnium olusatrum*	+	+	+	70

## Data Availability

The data presented in this study are available on request from the corresponding author, S.V.-N.

## Supplementary Materials

The following supporting information can be downloaded at https://www.mdpi.com/article/10.3390/plants15050766/s1, Figure S1: Localities in the Iberian Peninsula where the seeds of the 16 studied species were collected; Table S1: Collection data for the studied species, soil type and habitat description. Seeds and plants vouchers are in the MA Herbarium and the BGVMA Seed Bank at the Real Jardín Botánico, CSIC, Madrid.
