# Supplementary material for: Towards More Resilient Urban Landscapes: Optimal Sowing Season of 16 Native Mediterranean Species for Planting Designs
Source: Plants (Basel). 2026 Mar 2;15(5):766. doi: 10.3390/plants15050766 (PMC12986880; doi:10.3390/plants15050766)
Supplement: Supplementary file 1 [file plants-15-00766-s001.zip › plants-3990625-supplementary.pdf]

Table S1 Collection data for the studied species, soil type and habitat description. Seeds and plants vouchers are in the MA Herbarium and the BGVMA Seed Bank at the Real Jardín Botánico, CSIC, Madrid.

**Family**	**Collection number**	**Species**	**Date collection**	**Province**	**Locality**	**Altitude (m)**	**Geographical coordinates**	**Soil type**	**Habitat**
Apiaceae	SV160	*Bupleurum rigidum* L.	3.X.2020	Cuenca	Castillejo de la Sierra	957	40°22'37.85"N 2°9'33.86"W	Gravel and clays	Mediterranean forests and scrublands on all type of substrates
Asteraceae	LM10630	*Centaurea prolongoi* Boiss. ex DC.	13.VI.2020	Málaga	El Cascajal	609	36°36'32.72"N 4°37'20.36"W	Dolomite-crystalline	Rocky outcrops, roadsides, in clearings of pine forest or scrublands, on basic soils, slightly nitrified
Asteraceae	LM10223	*Centaurea clementei* Boiss. ex DC.	11.VI.2019	Cádiz	Zahara de la Sierra	580	36°50'19.83"N 5°23'24.39"W	Limestone	On highly exposed calcareous rocky outcrops and cliffs
Asteraceae	UAL	*Centaurea barrasii* Pau	21.V.2019	Almería	Carboneras	92	36°57'00.0"N 1°54'18.0"W	Calcareous	Esparto grassland and thyme scrublands on abandoned agricultural soils
Asteraceae	LM11063	*Cynara baetica* (Spreng.) Pau	20.IX.2020	Albacete	Riópar	748	38°25'22.65"N 2°32'15.17"W	Calcareous	Roadsides, fallow fields, forest clearings, nitrified soils
Asteraceae	LM10604	*Cynara humilis* L.	12.VI.2020	Málaga	Los Cerrillos	407	36°48'49.46"N 5°19'42.91"W	Calcareous	Roadside , fallows land and wasteland, forest and scrub clearings, on nitrified soils
Cistaceae	SV134	*Helianthemum squamatum* (L.) Dum. Cours.	28.VI.2019	Madrid	Morata de Tajuña	626	40°12'22.68"N 3°24'50.60"W	Gypsum	Open shrubland on gypsum-rich soils
Scrophulariaceae	LM10633	*Linaria clementei* Haens.	13.VI.2020	Málaga	El Cascajal	501	36°36'41.43"N 4°37'5.12"W	Dolomite-crystalline	Grasslands, scrublands, embankments, rocky outcrops near the road
Apiaceae	LM10489	*Magydaris panacifolia* (Vahl) Lange	20.VIII.2019	Madrid	Los Rancajales	947	40°44'7.90"N 3°45'32.92"W	Granite	Dry places, ditches, wastelands
Apiaceae	LM10491	*Margotia gummifera* (Desf.) Lange	20.IX.2019	Madrid	Lozoyuela	1124	40°56'37.09"N 3°38'10.19"W	Granite	Degraded scrublands
Brassicaceae	SV143	*Moricandia arvensis* (L.) DC.	3.V.2020	Madrid	Ensanche de Vallecas	621	40°22'00"N 3°36'41"W	Gypsum, loam	Disturbed sites

Lamiaceae	SV142	*Phlomis crinita* Cav.	1.VIII.2019	Almería	Sierra de Gádor	1732	36°52'18.19"N 2°46'04.57"W	Calcareous	Heliophilous scrublands
Lamiaceae	SV136	*Phlomis lychnitis* L.	02.VII.2019	Almería	Sierra de Gádor	743	36°49'52.10"N 2°50'47.27"W	Calcareous	Scrublands, near the road on nitrified sites
Apiaceae	LM10600	*Smyrnium olusatrum* L.	12.VI.2020	Cádiz	Zahara de la Sierra	527	36°50'27.07"N 5°23'21.15"W	Calcareous	More or less shady place on nitrified soils
Lamiaceae	SV137	*Thymus longiflorus* Boiss.	13.VII.2019	Granada	Sierra de Orce	1162	37°39'33.2"N 2°25'13.3"W	Calcareous	Scrub clearings, slopes and disturbed areas, in the understory of low-density pine forests.
Lamiaceae	SV138	*Thymus membranaceus* Boiss.	13.VII.2019	Almería	Sierra de María	1191	37°43'27.1"N 2°13'44.4"W	Gypsum	Scrub clearings, slopes and disturbed areas, in the understory of low-density pine forests. Gypsovag

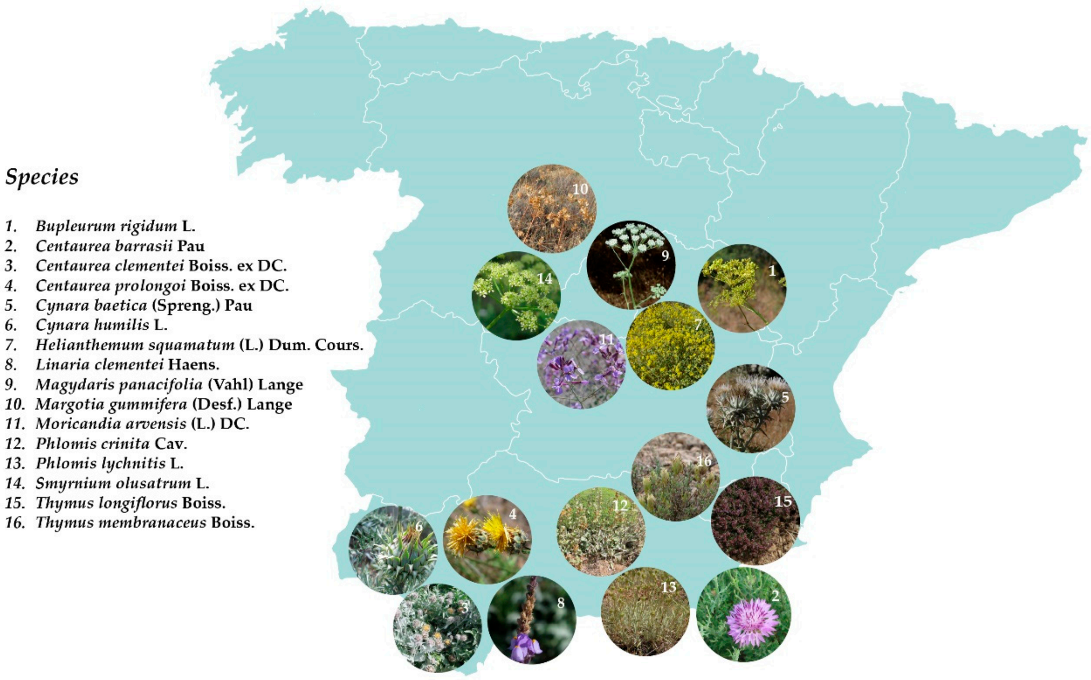

Figure S1 Localities in the Iberian Peninsula where the seeds of the 16 studied species were collected.
